# The Closeness Between Older Care Recipients and Family Caregivers Affects Caregivers’ Diet Habits

**DOI:** 10.1093/geroni/igaf122.2659

**Published:** 2025-12-31

**Authors:** Kumi Morishita-Suzuki

**Affiliations:** Sendai Center for Dementia Care Research and Practices, Sendai, Miyagi, Japan

## Abstract

In Japan, approximately 60% of family caregivers for older adults are aged ≥65 and face a higher risk of undernutrition due to caregiving burden. This study examined whether lower perceived closeness with care recipients is associated with poor dietary habits among caregivers. Data from Japan’s 2024 national family caregiver panel survey were analyzed, focusing on caregivers aged ≥65 (n = 496, mean age = 70.8, SD = 5.0). Poor dietary habits were defined as a Dietary Variety Score < 4, with self-rated closeness as the independent variable. Covariates included caregivers’ age, economic status, solitary eating, chronic diseases, relationship with the care recipient, level of care needed, dementia diagnosis, caregiver burden, and use of caregiving services. Binary logistic regression analysis was conducted. The study was approved by the research ethics committee (No. 24U01). Results showed that 15.4% of men and 17.7% of women reported low closeness with care recipients. Poor dietary habits were found in 42.2% of men and 24.4% of women. Multivariate analysis revealed no significant association between closeness and dietary habits in men. However, among women, lower closeness was significantly linked to poor dietary habits (odds ratio = 3.01, 95% CI [1.21, 7.54], p = .018). These findings suggest that women caregivers with lower perceived closeness to care recipients may be more vulnerable to poor dietary habits, whereas men, typically less involved in meal preparation, may be less affected. Future studies should explore how caregiver-recipient relationships influence appetite and food choices.

